# Editorial: Chagas disease novel drug targets and treatments

**DOI:** 10.3389/fcimb.2023.1199715

**Published:** 2023-05-26

**Authors:** Vilma G. Duschak, Alberto E. Paniz Mondolfi, Gustavo Benaim

**Affiliations:** ^1^ National Council of Scientific and Technical Research (CONICET) and National Institute of Parasitology (INP), “Dr.Mario Fatala Chaben”, Administración Nacional de Laboratorios de Institutos de Salud (ANLIS)-Malbrán, National Health Department, Ciudad Autónoma de Buenos Aires (CABA), Buenos Aires, Argentina; ^2^ Molecular Microbiology Laboratory, Department of Pathology, Molecular and Cell-Based Medicine, Icahn School of Medicine at Mount Sinai, New York, NY, United States; ^3^ Incubadora Venezolana de la Ciencia (IVC), Centro de Investigaciones Biomédicas IDB, Barquisimeto, Venezuela; ^4^ Unidad de Señalización Celular y Bioquímica de Parásitos, Instituto de Estudios Avanzados (IDEA), Caracas, Venezuela; ^5^ Instituto de Biología Experimental, Facultad de Ciencias, Universidad Central de Venezuela, Caracas, Venezuela

**Keywords:** Chagas disease, drug targets, treatment strategies, drug repurposing, neglected diseases

Chagas disease (ChD), also known as American Trypanosomiasis, is a parasitic disease caused by the hemoflagellate protozoan *Trypanosoma cruzi*. The disease is endemic to Latin America with an estimated 6-7 million people infected. Today, ChD is considered and emerging global health concern due to imported cases through traveling and migration, as well as to its broadening geographic endemism, like in the case of the United States (Paniz Mondolfi et al., 2020). Despite this, treatment options for ChD remain limited and exhibit significant adverse effects. Current treatment options are based on two nitroderivative and nitrofuran compounds, Benznidazole (Bz) and Nifurtimox (Nx), which were introduced in clinical medicine over 50 years ago and despite being the only two drugs approved for the treatment of ChD, present several limitations for their use.

First, both drugs Bz and Nx exhibit significant side effects, ranging from mild to severe systemic signs and symptoms, including skin rashes, nausea, vomiting, anorexia, anemia, leukopenia and peripheral neuropathy, that usually lead to discontinuation of treatment. Second, the efficacy of these drugs varies depending on the developmental phase of the parasite, stage of the disease (acute or chronic) and the geographical location of the patient, with cure rates ranging from 60 to 80%. This is important, because geographic location is intimately related to the genomic variability of the parasite and its respective Discrete Typing Units (DTUs) which are known to exhibit differential trends in response to treatment (Higuera et al., 2013). Thirdly, the duration of therapy is prolonged, ranging from 60 to 120 days, requiring close monitoring due to potential adverse effects. Lastly, the emergence of drug-resistant strains of *T. cruzi* has represented a major obstacle to the successful treatment of ChD. Hence, there is an urgent need to identify new drugs and drug targets to improve the efficacy and safety profile of ChD therapy.

As a result, significant efforts are being made in search for novel chemotherapeutic approaches for this invariably inveterate infection, particularly at its chronic phase, the most insidious and frequent clinical expression of the disease. Therefore, breaking ground in Chagas disease treatment, this topic is unveiling novel drug targets and drug repurposing strategies. The increase in the understanding of the biology and biochemistry of the parasite have allowed the identification of new therapeutic targets to identify new trypanocidal agents, in addition to rational drug design and screening of natural products. Existing drugs are active in treating congenital infection by *T. cruzi*, acute ChD, and children up to 12 years, without any evidence for its effectiveness of treatment in adults at the chronical (and more frequent) phase of the infection. Therefore, novel active trypanocidal compounds, with low toxicities and increased efficacies during the chronic phase as stated above are immediately required and reviewed in detail (Duschak, 2011; Duschak, 2016; Duschak, 2017). In the last years, different types of parasite targets have been classified in three main groups based in those that most researchers are working on (Duschak, 2019). One of the main approaches on the search for new drugs consists in exploiting biological differences between the parasite and the mammalian host-cells. For example, since the early recognition that these parasites possess ergosterol at their membranes as a final products of sterol synthesis (sharing this property with fungi), instead of cholesterol (which is characteristic of mammalian cells), ergosterol synthesis have shown to be potentially candidates for a rational therapeutic approach (Urbina et al., 1998).

Another interesting approach would base itself in drug repurposing and combination therapy, which represent potentially cost-effective strategies. In these lines, Miltefosine (Milt), a synthetic alkyllysophospholipid initially developed for treatment of breast cancer and successfully repurposed for oral treatment of leishmaniasis (Croft et al., 1996), has been proposed as a promising candidate against *T. cruzi* infection (Rodriguez-Duran et al., 2019; Benaim et al., 2020).

Interestingly, its mechanism of action on trypanosomatids has been recently elucidated (Pinto-Martinez et al., 2018; Rodriguez-Duran et al., 2019). In fact, one of the papers included in this collection, *“Miltefosine and Benznidazole combination improves anti-T. cruzi in vitro and in vivo efficacy’*, precisely touches on the efficacy of Milt monotherapy as well as in combination with Bz, both in *in vitro* and *in vivo* models of *T. cruzi* infection. It is worth noting that the results described here support the efficacy of Milt’s activity in clinically relevant stages of *T. cruzi* infection and lend further support for evaluating combined schemes using Milt and other synthetic alkyllysophospholipids as likely drug candidates (Gulin et al.).

Along the lines of drug-repurposing candidates are the benzofuran derivatives, specifically amiodarone and dronedarone. Amiodarone, a commonly prescribed antiarrhythmic in Chagas cardiomyopathy as an antiarrhythmic, has shown promising effects against *T. cruzi* and other trypanosomatids (Benaim et al., 2021) either as monotherapy or in combination with sterol biosynthesis inhibitors, such as posaconazole and itraconazole, acting in a synergistic mode (Benaim et al., 2006). The combination of amiodarone and itraconazole (a triazole from which posaconazole derived) has proved successful in treating naturally infected dogs, resulting in the parasitological cure of all animals tested in what is considered the largest investigational drug trial for ChD performed in dogs to date (Madigan et al., 2019).

The work presented by Almeida-Silva et al. conducted a study using the repurposed drug disulfiram (DSF) and its derivative diethyldithiocarbamate (DETC) in combination with Bz to treat *T. cruzi* both *in vitro* and *in vivo*. Their findings demonstrated that the DETC-Bz combination exhibited a synergistic effect, reducing epimastigotes proliferation and increasing selective indexes over a 10-fold. Further evidence revealed through electron microscopy imaging revealed membrane discontinuities, cell body volume reduction, and significant enlargement of endoplasmic reticulum cisternae. Additionally, dilated mitochondria with decreased electron density and disorganized kinetoplast DNA were observed substantiating the effects of this combination. According to the authors, the combination of DSF and DETC has the potential to reduce the toxicity of other drugs as well as the emergence of drug resistance phenotypes, making it a safe and effective option for ChD treatment.

The studies presented in this collection also provide important insights on other emerging therapeutic strategies. As an example, novel structural data on mitochondrial *T. cruzi* peroxiredoxin (PRXs), expressed in all stages of the parasite, central for the survival and replication of the parasite, have been proposed as virulence factors which detoxify oxidizing agents, central for the survival and replication of the parasite have been considered as possible therapeutic targets but there is still no specific drug against them. Structural data for mitochondrial *T. cruzi* peroxiredoxin as novel target were compared with several PRXs showing high similarity with human peroxiredoxin 3, paves the way for exploiting similar targets with enhanced efficacy. Additionally, the antibiotic Thiostrepton has also been considered as a promising potential inhibitor molecule as trypanocidal drug within the frame of drug repurposing. The results have also demonstrated a synergic effect of Thriostepton and Bz (Rivera Santiago et al.).

Here, the role of ivermectin as a potential trypanocidal drug against *T. cruzi* and other trypanosomatids was also investigated. Ivermectin, was found to affect the proliferation of different *T. cruzi* developmental stages (epimastigotes, amastigotes, and trypomastigotes) in a dose-dependent manner. At 50 μM, the drug had a trypanostatic effect on the epimastigote stage, while at 100 μM, it exhibited trypanocidal effects. Moreover, combination treatment of ivermectin with Bz or Nx demonstrated important synergistic effects (Fraccaroli et al.).This pan-stage targeted coverage of ivermectin against *T. cruzi* is without doubt a promising finding that would allow the potential to treat ChD all across its clinical spectrum, which are governed by different life cycle stages of the parasite.

A related paper to this topic explored the role of Na^+^/Ca^2+^ exchange (NCX) in C57BL/6 *T. cruzi* Y strain infected mice. Two NCX blockers, KB-R7943 and YM-244769, were found to reduce diastolic Ca^2+^ concentration ([Ca^2+^]_d_) in cardiomyocytes during the early acute, acute, and chronic phases, and prevented the increase in ([Ca^2+^]_d_) associated with exposure to a Na^+^-free solution. These findings suggest that Ca^2+^entry through NCX in reverse mode plays a significant role in the observed disrupted [Ca2+]d homeostasis in infected cardiomyocytes. Furthermore, NCX inhibitors may be a viable therapeutic approach for treating patients with ChD cardiomyopathy, thus improving therapeutic options for the disease (Lopez et al.).

Another important aspect of ChD pathogenesis relates to immunity against the parasite. The immune response to *T. cruzi* is complex and involves action of both, the innate and adaptive immune components. However, within the promising approaches currently being developed for ChD therapy, immunomodulatory drugs and compounds play an important role, and some have already shown to enhance the immune response to *T. cruzi* and promoting parasite clearance. Along these lines, this collection also includes several pieces dealing with important aspects not only on the innate and adaptive immune responses responsible for controlling infection and preventing disease progression, but also on the potential immunomodulatory effects of several compounds such as PepA and fenofibrate.

For example, the synthetic peptide, PepA, containing the CTHRSSVVC sequence, is a mimicker of the CD163 and TNF-α tripeptide “RSS” motif, which binds to atheromatous plaques in carotid biopsies of human patients, spleen tissues, as well as low-density lipoprotein receptor knockout (LDLr−/−) mouse model of atherosclerosis. Here, the potential theranostic role of PepA was investigated by studying its effect on experimental models of *T. cruzi* infection both *in vitro* and *in vivo*. Findings of this study demonstrated that PepA and PepB, a peptide with a random sequence, reduced the intracellular parasitism of peritoneal mouse macrophages but were inactive during cardiac cell infection. However, PepA and PepB did not display trypanocidal effects on bloodstream trypomastigotes and did not exhibit *in vivo* efficacy when administered after parasite inoculation. The *in vitro* activity of PepA and PepB on the infection of peritoneal mouse macrophages by *T. cruzi* was reported, as possibly triggering the microbicidal arsenal of host professional phagocytic cells, capable of controlling parasitic invasion and proliferation (Leite et al.).

On the other hand, fenofibrate was shown to increase the population of non-classical monocytes in asymptomatic ChD patients, and to modulate inflammatory cytokines in PBMCs. Chronic ChD cardiomyopathy is the most relevant clinical manifestation of ChD infection. In previous studies, fenofibrate, a PPARα agonist, has shown to control inflammation, prevent fibrosis, and improve cardiac function in a murine infection model. In the current study, the authors investigated the spontaneous release of inflammatory cytokines and chemokines, changes in the frequencies of monocyte subsets, and the effects of fenofibrate on PBMCs of seropositive patients amongst different clinical stages of ChD. Their findings suggest a potential therapeutic role for fenofibrate as a modulator of monocyte subpopulations towards an anti-inflammatory profile in different stages of chronic ChD (Pieralisi et al.). Also, regarding the role of the complement system in the modulation of T-cell responses in chronic Chagas disease a minireview has been presented in this Topic (Albareda et al.).

Drug discovery, remains an important component in the quest for ChD therapy. An example of a most recent breakthrough was the discovery of the orally active benzoxaborole prodrugs which have proved effective not only against the parasite but against the various *T. cruzi* lineages (DTUs) described to date (Kingwell, 2022; Padilla et al., 2022). In this collection, an interesting *in silico* work by Ros-Lucas et al., used the AlphaFold Protein Structure Database, which homes 19,036 protein models from *T. cruzi*, to demonstrate not only key functions on describing new therapeutic approaches, but also to shed light on molecular mechanisms of action for known compounds. In what they call “proof-of-concept study”, they screened the AlphaFold *T. cruzi* set of predicted protein models to find prospective targets for a pre-selected list of compounds with known anti-trypanosomal activity using docking-based inverse virtual screening. The results obtained in this work provide insight into the mechanisms of action of the compounds and their targets, and points to new strategies to finding novel compounds or optimize already existing ones.

Understanding the physio-pathological substrates of disease is of extreme importance to understand how components of the innate immune and adaptive immune system intervene and modulate disease progression. Here, Caputo et al. explore the intricacies of the interaction between the complement system and the T cell response and discuss on the potential role that anaphylatoxins (C3a and C5a) might play in T cell responses during chronic human *T. cruzi* infection. Also herein, Rodrigues Ferreira et al. also discuss on the role that transforming growth factor beta (TGF-β) signaling pathway on *T. cruzi*-infection and its biological implications. The authors found that addition of the 1D11 monoclonal antibody to cardiac cells greatly reduced *T. cruzi* cardiomyocyte invasion. In addition, the authors also demonstrated most importantly that treatment with 1D11 reduced cardiac fibrosis and reversed electrical abnormalities improving cardiac performance. The latter finding is of utmost importance, because it validates a two-target approach in treating the disease, not only by directly reducing parasitemia but also by modulating host component promoting tissue repair. This “killing two birds with one stone” strategy is also the mainstay for Amiodarone’s’ broad mechanism of action in treating Chagas cardiomyopathy (Benaim and Paniz Mondolfi, 2012).

Amiodarone acts through a dual mechanism of action, not only aiding in the recovery of cardiac function but also by exerting specific anti-*T. cruzi* effects, based on the drugs ability to disrupt the parasite’s Ca^2+^ homeostasis, claimed to be an important target of drug action in these parasites (Benaim et al., 2020) and by blocking *de novo* ergosterol biosynthesis in its membrane (Benaim et al., 2006). Additionally, amiodarone promotes cardiac cell recovery by inducing reassembling of cytoskeleton elements and re-establishing essential gap protein communication such as connexin 43 between myocardiocytes, which are essential to re-establish cardiac contractility and control arrythmogenic events (Adesse et al., 2008; Adesse et al., 2011). This is why Amiodarone remains as the archetype examples of drug repurposing in ChD given its dual role, not only as an antiarrhythmic drug, but also as an antiparasitic agent (Benaim and Paniz Mondolfi, 2012).

Chagas disease (ChD) remains a major public health concern in Latin America, where it is endemic, and its prevalence is expanding globally, including the United States. The limited treatment options for ChD, based on the old nitroimidazole and nitrofuran derivatives Benznidazole (Bz) and Nifurtimox (Nx), have significant limitations in terms of efficacy, side effects, and duration of the treatment. There is an urgent need for novel drugs and therapeutic strategies to improve the efficacy and safety profile of ChD therapy. The collection of studies presented here highlights several promising approaches, including drug repurposing and combination therapy, targeting ergosterol synthesis inhibitors, benzofuran derivatives, mitochondrial *T. cruzi* peroxiredoxin and the use of macrocyclic lactones (ivermectin) and the alkylphosphocholine drug Miltefosine as potential therapeutic agents. We also emphasize on the role of key innate and adaptive immune components, as well as the immunomodulatory effects of Fenofibrate and potential for monoclonal antibody therapy. These approaches, along with others presented here, offer hope to those affected by this neglected infectious disease.

## Author contributions

VG has contributed editing 8 articles. AM edited 3 articles. GB has contributed to the edition of 2 articles. The contribution among co-editors has been interactive during the whole process. All authors contributed to the article and approved the submitted version.
